# Development of Type 1 Diabetes may occur through a Type 2 Diabetes mechanism

**DOI:** 10.3389/fendo.2022.1032822

**Published:** 2022-12-14

**Authors:** Knud Josefsen, Lars Krogvold, Ivan C. Gerling, Flemming Pociot, Knut Dahl-Jørgensen, Karsten Buschard

**Affiliations:** ^1^ The Bartholin Institute, Department of Pathology, Rigshospitalet, Copenhagen Biocenter, Denmark; ^2^ Division of Pediatric and Adolescent Medicine, Oslo University Hospital, Oslo, Norway; ^3^ Faculty of Medicine, University of Oslo, Oslo, Norway; ^4^ Department of Medicine, University of Tennessee, Memphis, TN, United States; ^5^ Faculty of Health and Medical Sciences, University of Copenhagen, Copenhagen, Denmark

**Keywords:** type 1 diabetes, type 2 diabetes, pathogenesis, beta cells, GLP-1, metformin

## Abstract

**Background:**

At diagnosis of Type 1 Diabetes (T1D), 30% of the beta cells are dormant, i.e. alive, but inactive. This could reduce beta cell destruction, as cellular stress contributes to beta cell damage. However, the beta cells, that are still active, must produce more insulin and are therefore more vulnerable. The inactive beta cells represent a potential for restoring the insulin secretion.

**Methods:**

We analyzed the expression of selected genes in islets from live, newly diagnosed T1D patients from the DiViD study and organ doners with longer duration of T1D, type 2 diabetes (T2D), or no diabetes from the nPOD study. Additionally, analysis of polymorphisms was performed on all the investigated genes.

**Findings:**

Various possibilities were considered for the inactivity of the beta cells: secretion defect, fetal state, hibernation, and insulin resistance. We analyzed genes related to the ceramide and sphingomyelin synthesis and degradation, secretion, circadian rhythm and insulin action, and found changes in T1D islets that resemble fetal dedifferentiation and asynchrony. Furthermore, we found low levels of insulin receptor mRNA in the islets. No polymorphisms were found.

**Interpretation:**

Our findings suggest a secretion defect, but also fetal dedifferentiation and desynchronization in the inactive beta cells. Together with previous evidence, that predisposing factors for T2D are also present for T1D development, we raise the idea to treat individuals with ongoing T1D development prophylactically with T2D medicine like GLP-1 receptor agonists, metformin, or others, combined with anti-inflammatory compounds, in order to reactivate the dormant beta cells, and to prevent autoimmune destruction. T2D mechanisms during T1D development should be investigated further.

## Introduction

In the DiViD program (1) with biopsies from patients with newly diagnosed T1D, it has been found that 60% of the beta cells have been destroyed in the ongoing insulitis process, approximately 30% of the beta cells are preserved, but inactive, and approximately 10% actively secrete insulin for maintaining glucose homeostasis (2). However, these few remaining, actively secreting beta cells, must be considered at high risk of immune destruction for several reasons: the cells express high amounts of antigens (3), show ER-stress (4), produce high levels of immunogenic insulin molecules (5), express TTG (tissue trans-glutaminase), which deamidates glutamine to the more risky glutamate (6), and are more sensitive to cytokines (7). Overall, T1D development is seen more frequently in individuals with stressed beta cells, as is seen during the third trimester of pregnancy (8), during virus infections (9), and in association with psychological stress. It might be avoided, at least in animal models, by prophylactic insulin treatment (10), and in individuals with T1D, C-peptide values often increase substantially the first month after a T1D diagnose, when insulin injections are instituted. In humans, prophylactic insulin treatment has not been demonstrated to be efficient (11), possibly due to suboptimal insulin dosage.

A large fraction of the beta cells in the pancreas is inactive at the time of diagnosis. From an individual cell’s perspective, this is appropriate since the cell might be shielded from the destructive forces that attack active cells. The disadvantage is that higher secretory activity, and thus a higher risk of immune attack, is placed on the remaining, active beta cells.

The questions we address in this study are how the beta cells become dormant, and what can be done to prevent the T1D process in reaching clinical disease. We have analyzed islet mRNA expression from the DiViD and nPOD studies to suggest answers. Two of the possible reasons for the inactivity could be that the beta cells enter a fetal mode or a state of hibernation.

## Methods

### RNA analysis

Human pancreatic tissue was from the DiViD study (12) from newly onset (disease duration 35 days) T1D patients aged 24–35 years (n=5), or from the nPOD study (13): autoantibody-positive (n=12), T1D patients of 5 [0-21] years diabetes duration (n=20), T2D patients with 2 [0-15] years duration (n=8), pancreas-transplanted T1D patients with failure of the transplant (n=4), and healthy controls (n=18). The DiViD study was approved by The Norwegian government’s Regional Ethics Committee (reference 2009/1907) and the nPOD study by the University of Tennessee Health Science Center’s Local Institutional Review board (reference 10-00848-XM). Direct to lars.krogvold@gmail.com for requests to the datasets. In contrast to the living patients in the DiViD study, the nPOD study consists of pancreases from organ donors following accidental death. Frozen sections were used for laser capture dissection (14) and islets from two to five sections were pooled, and RNA was extracted, using the Arcturus PicoPure RNA Isolation Kit (Applied Biosystems, Grand Island, NY, USA), and quantified on a Bioanalyzer 2100 instrument (Agilent Technologies, Santa Clara, CA, USA). Gene expression analysis was carried out using Affymetrix expression arrays (GeneChip Human Gene 2.0 ST, Thermo Fisher) and normalized using global scaling (15). To maximize comparison, all tissue handling was performed in the same laboratory, by the same technicians, analyzed on the same equipment, and the RNA quality was certified by RIN value measurements (> 3.5).

### GWAS analysis

GWAS analysis included the genes shown in **Table 2**. SNPs were obtained from Onengut-Gumuscu et al (16), using a cut-off p value <0.01. SNPs within ±250 kb flanking regions of the transcription start site of the examined genes were identified, and these SNPs, that were likely to regulate the expression of the associated genes, were identified using Encyclopedia of DNA Elements (ENCODE) from the University of California 186 Santa Cruz genome browser (17) (http://genome.ucsc.edu/), RegulomeDB (18), and data from multiple expression quantitative trait locus (eQTL) studies (19). The cis-eQTL effects were calculated using linear regression models in the selected tissues. Validated eQTLs from Westra et al. (20), and GTEx2015_v6. GTEx2015_v6 eQTLs were computed using a ±1 Mb cis window around the transcription start site. Significance was determined using a Q value threshold, using ≥ 70 samples per tissue to achieve the statistical power needed for this type of analysis. Predicted eQTL was calculated for pancreas and whole blood. Genotype–tissue expression predictions calculation was performed in tissues with at least ten samples. No Q value filtering was performed.

### Statistical analysis

Statistical analysis for RNA expression was performed using GraphPad Prism 8.0.2 (GraphPad, La Jolla, Ca, USA) and data are shown as mean ± SEM. Outliers were detected in each group using the ROUT method and a total of 21 data points across all genes and groups were identified and removed. All groups were tested for normal distribution by the D’Agostino–Pearson and Anderson Darling test. For comparison between groups, one-way ANOVA was used with Dunnett’s multiple comparison test and a 95% CI. P <0.05 was considered significant (shown as *p < 0.05, **p < 0.01 and ***p < 0.001 in the figures).

## Results

**Figure 1:**
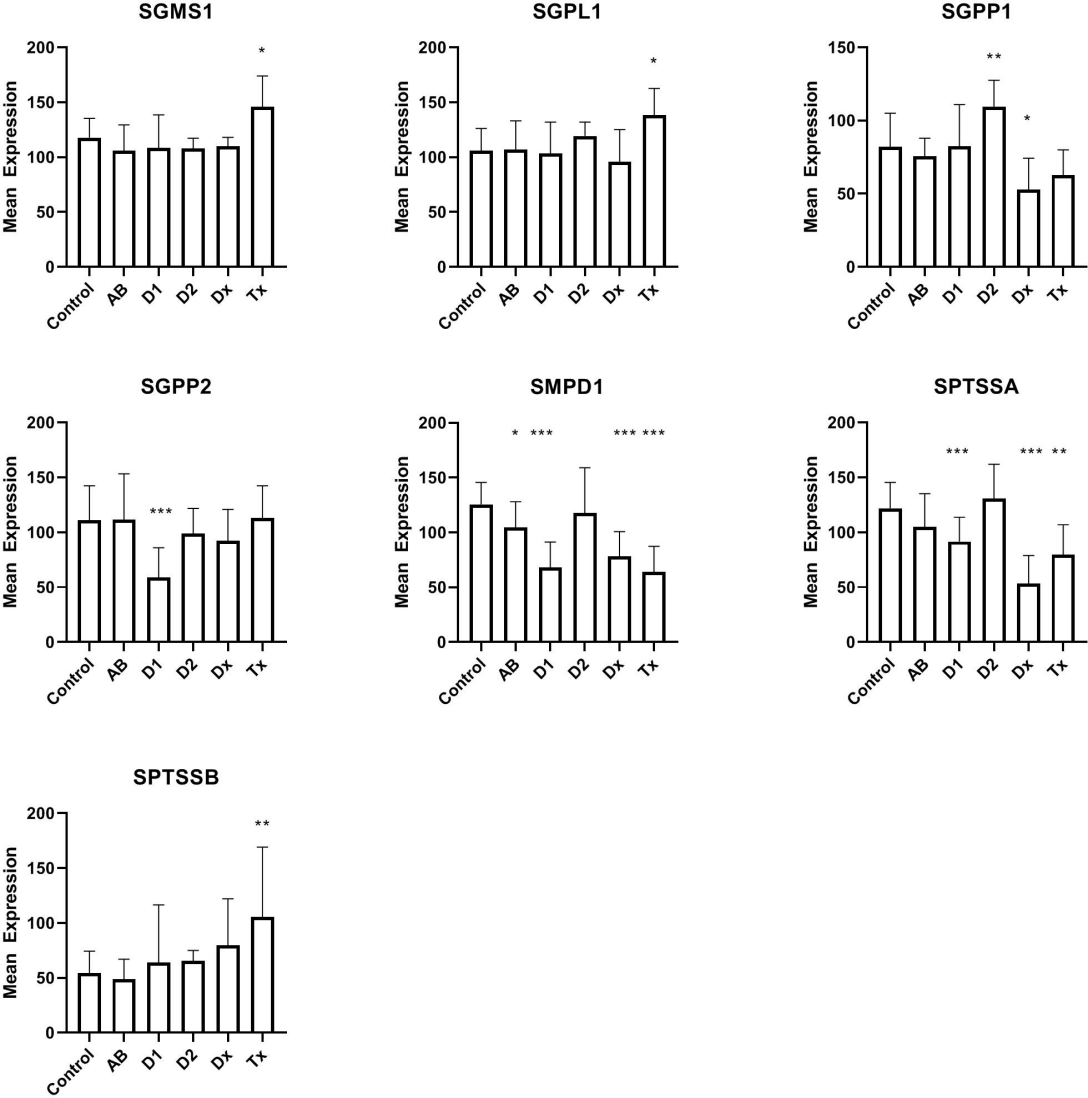
Expression levels of islet of Langerhans mRNA from genes related to synthesis or degradation of ceramide or sphingomyelin. For comparison between groups, one-way ANOVA was used with Dunnett’s multiple comparison test and a 95% CI. C: non-diabetic control patients, AB: non-diabetic subjects with auto-antibodies, D1, Type 1 diabetes of longer duration; D2, Type 2 diabetes; Dx, Newly diagnosed Type 1 DiViD samples; Tx, Biopsies from pancreatic transplants with recurrent diabetes. *p<0.05, **p<0.01, ***p<0.001.

In the DiViD and nPOD material (see patient characteristics in **Table 1**) we initially looked at expression of genes (**Figure 1**) coding for enzymes that participate in the maintenance and degradation of ceramide and sphingomyelin and that were not previously examined (21). Interestingly, SMPD1 (also called ASM, acid sphingomyelinase) which degrades sphingomyelin, is reduced in all patient groups related to T1D. This is also the case for SPTSSA, although the reduction in antibody-positive individuals does not reach statistical significance. SGPP1 is also reduced at the diagnosis of T1D, whereas SGPP2 is only reduced in long term T1D individuals. SGMS1, SGPL1, and SPTSSB showed increased expression only in transplanted islets with recurrent diabetes (Tx). Tissue from T2D donors does not show changes in gene expression, except for SGPP1, where the gene is oppositely regulated than in tissue from T1D patients. In addition to the genes in **Figure 1**, ACER1, ACER3, CEPT1, CHPT1, and PCYT1A (see **Table 2** for gene names), were also examined, but were not differentially regulated.

**Table 1:** Characteristics for the investigated patient groups. Below is shown identification number related to DiViD and nPOD repositories of analyzed samples.

		n (M/F)	Age	BMI	Diabetes duration	C-peptide
Controls		18 (9/9)	35.5 [14.2-68]	25.1 [14.9-35.1]		2.9 [0.5-17.9]
T1D	Long term T1D	20 (10/10)	19 [5-43.5]	24.6 [11,9-30.9]	5 [0-21] y	0.05 [0.05-0.48]
Ab	Antibody positive	12 (7/5)	38.5 [4.3-69.2]	25.4 [14.8-34.3]		5.4 [0.06-26.2]
T2D	Type 2 diabetes	8 (3/5)	43.9 [18.8-62]	35.7 [19.9-41]	2 [0-15] y	3.5 [0.58-10.7]
Dx	T1D at diagnosis	5 (3/2)	31 [24-35]	25.6 [20,9-28.6]		NA
Tx	Transplanted, recurrent	4 (3/1)	44.5 [38-63]	23.9 [22.7-26]		NA

**Controls** 6013, 6024, 6048, 6075, 6012, 6099, 6140, 6162, 6168, 6227, 6129, 6165, 6102, 6229, 6251, 6010, 6179, 6019

**T1D** 6070, 6084, 6088, 6180, 6224, 6243, 6228, 6209, 6038, 6046, 6069, 6195, 6052, 6268, 6265, 6196, 6211, 6113, 6264, 6135

**Ab** 6080, 6123, 6158, 6167, 6171, 6044, 6101, 6154, 6156, 6181, 6197, 6147

**T2D** 6188, 6114, 6249, 6275, 6273, 6191, 6059, 6110

**Dx** DiViD-6, DiViD-5, DiViD-4, DiViD-3, DiViD-2

**Tx** 3678-01, 3681-02, 3717-01, 3626-D

**Table 2:** List of abbreviations of examined genes and sample numbers from the nPOD and DiViD repository.

*AANAT*	aralkylamine N-acetyltransferase
*ACER1*	alkaline ceramidase 1
*ACER3*	alkaline ceramidase 3
*AKT1*	AKT serine/threonine kinase 1
*ARNTL*	aryl hydrocarbon receptor nuclear translocator like
*ARNTL2*	*BMAL2*, Basic Helix-Loop-Helix ARNT Like 2
*ASMT*	Acetylserotonin O-Methyltransferase
*CEPT1*	choline/ethanolamine phosphotransferase 1
*CHPT1*	choline phosphotransferase 1
*CLOCK*	clock circadian regulator
*CRY1*	Cryptochrome Circadian Regulator 1
*CRY2*	cryptochrome circadian regulator 2
*DBP*	D-Box Binding PAR BZIP Transcription Factor
*FOXO1*	Forkhead Box O1
*GPR50*	G Protein-Coupled Receptor 50
*HES1*	hes family bHLH transcription factor 1
*IGF1*	insulin like growth factor 1
*IGF2R*	insulin like growth factor 2 receptor
*IL6*	interleukin 6
*INSR*	insulin receptor
*MTNR1A*	Melatonin Receptor 1A
*MTNR1B*	Melatonin Receptor 1B
*NPAS2*	Neuronal PAS Domain Protein 2
*NR1D1*	Nuclear Receptor Subfamily 1 Group D Member 1
*NR1D2*	nuclear receptor subfamily 1 group D member 2
*PASK*	PAS domain containing serine/threonine kinase
*PCYT1A*	phosphate cytidylyltransferase 1A
*PDX1*	pancreatic and duodenal homeobox 1
*PER1*	Period Circadian Regulator 1
*PER2*	period circadian regulator 2
*PER3*	period circadian regulator 3
*PPARG*	peroxisome proliferator activated receptor gamma
*RORA*	RAR related orphan receptor A
*SGMS1*	sphingomyelin synthase 1
*SGPL1*	sphingosine-1-phosphate lyase 1
*SGPP1*	sphingosine-1-phosphate phosphatase 1
*SGPP2*	sphingosine-1-phosphate phosphatase 2
*SIRT*	sirtuin
*SMPD1*	sphingomyelin phosphodiesterase 1
*SOX9*	SRY-box transcription factor 9
*SPTSSA*	serine palmitoyltransferase small subunit A
*SPTSSB*	serine palmitoyltransferase small subunit B
*SYT11*	synaptotagmin 11
*SYT13*	synaptotagmin 13
*SYT16*	synaptotagmin 16
*SYT4*	synaptotagmin 4
*SYT7*	synaptotagmin 7
*SYT9*	synaptotagmin 9
*TSC22D3*	TSC22 Domain Family Member 3
*TSPAN4*	Tetraspanin 4
*USP2*	ubiquitin specific peptidase 2

**Figure 2:**
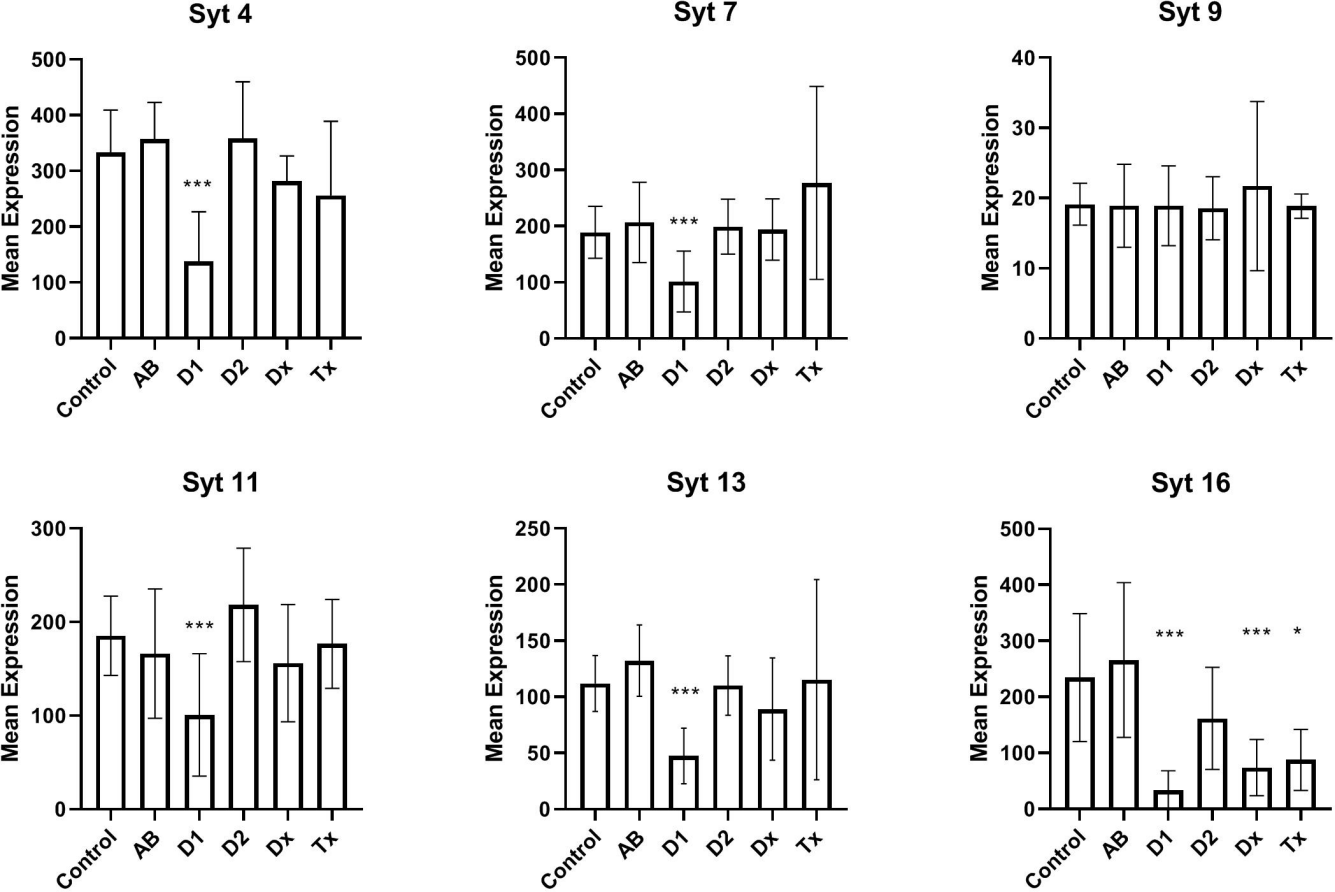
Expression levels of selected synaptotagmins in human islet of Langerhans. See **Figure 1** for patient groups and additional information. *p<0.05, **p<0.01, ***p<0.001.

In **Figure 2** we have analyzed the expression of selected synaptotagmins to investigate the hypothesis that the inactive state of many beta cells could simply be a result of a malfunctioning exocytotic mechanism. Thus, synaptotagmins, some of which binds Ca^++^, are assumed to be regulators of secretion, primarily in neuronal cells, but they have also been found in pancreatic beta cells. We found that synaptotagmin 4,7,11,13,16 are downregulated in long-standing T1D patients, whereas in newly diagnosed (DIVID) T1D patients only synaptotagmin 16 is downregulated.

**Figure 3:**
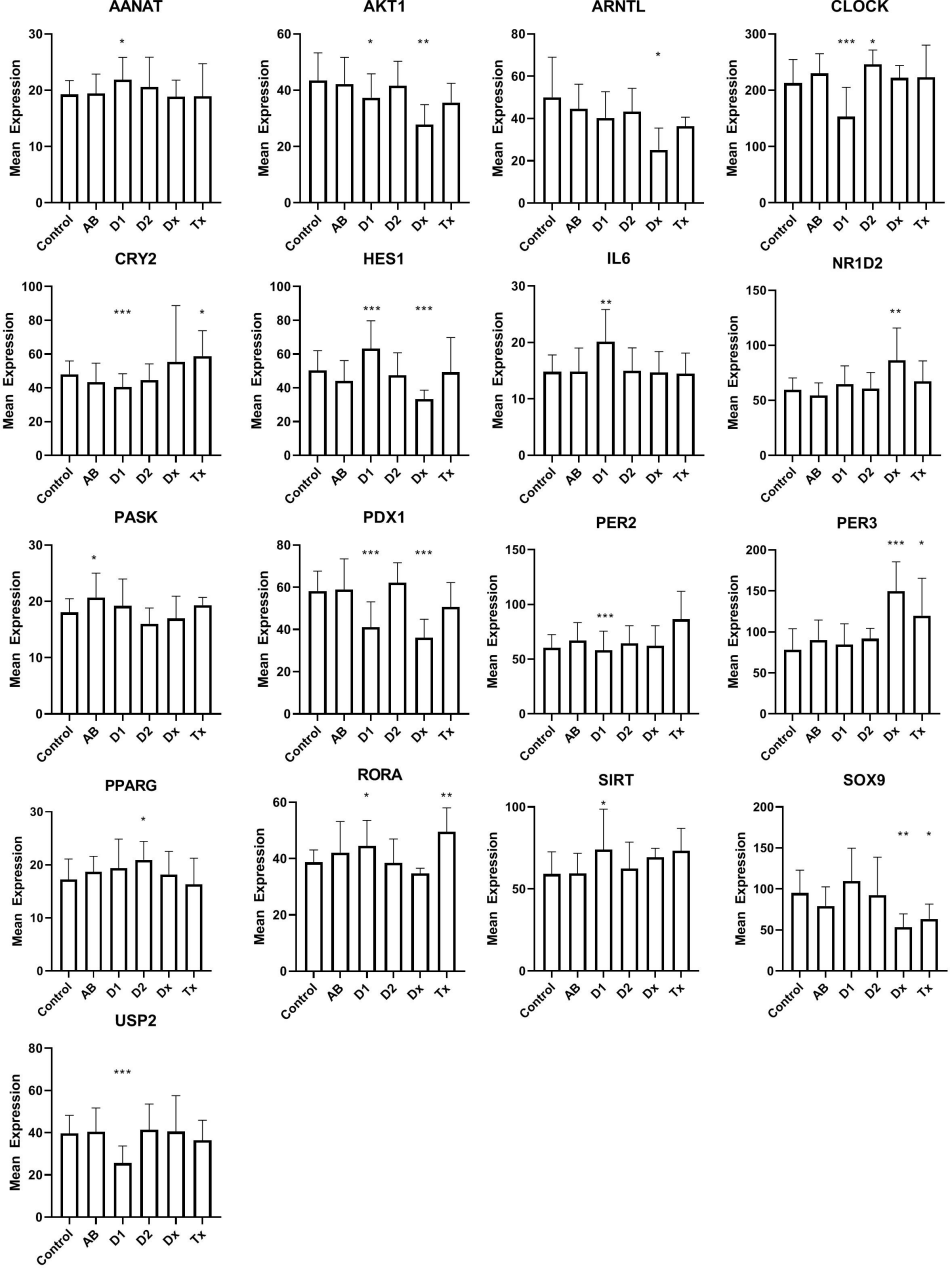
Expression levels of selected genes related to circadian rhythm in islet of Langerhans. See **Figure 1** for patient groups and statistics. *p<0.05, **p<0.01, ***p<0.001.

Dormant cells are well known in the animal kingdom during hibernation. This state, that enables species to overcome environmental changes, that is otherwise not compatible with their survival, is only partially understood, but changes in clock genes, otherwise known to control the circadian rhythm, can be detected in many organs during this state. This led us to examine if the inactive beta cells, at the time of T1D diagnosis, could bear resemblance to hibernation by investigating the expression of clock genes (**Figure 3**). Of 31 investigated genes, that were selected, either because they have been demonstrated as temporally regulated in beta cells or are genes that are well known to regulate the circadian rhythm in cells, 16 genes were differentially regulated, 10 in long lasting T1D, and 7 in tissue from T1D patients at the time of diagnosis (**Figure 3**). Only 2 genes were differentially regulated in islet tissue from T2D patients compared with controls. We did not find changes in NPAS2, PER1, CRY1, FOXO1, NR1D1, ARNTL2, DBP, TSC22D3, TSPAN4, ASMT, MTNR1A, MTNR1B, or GPR50.

**Figure 4:**
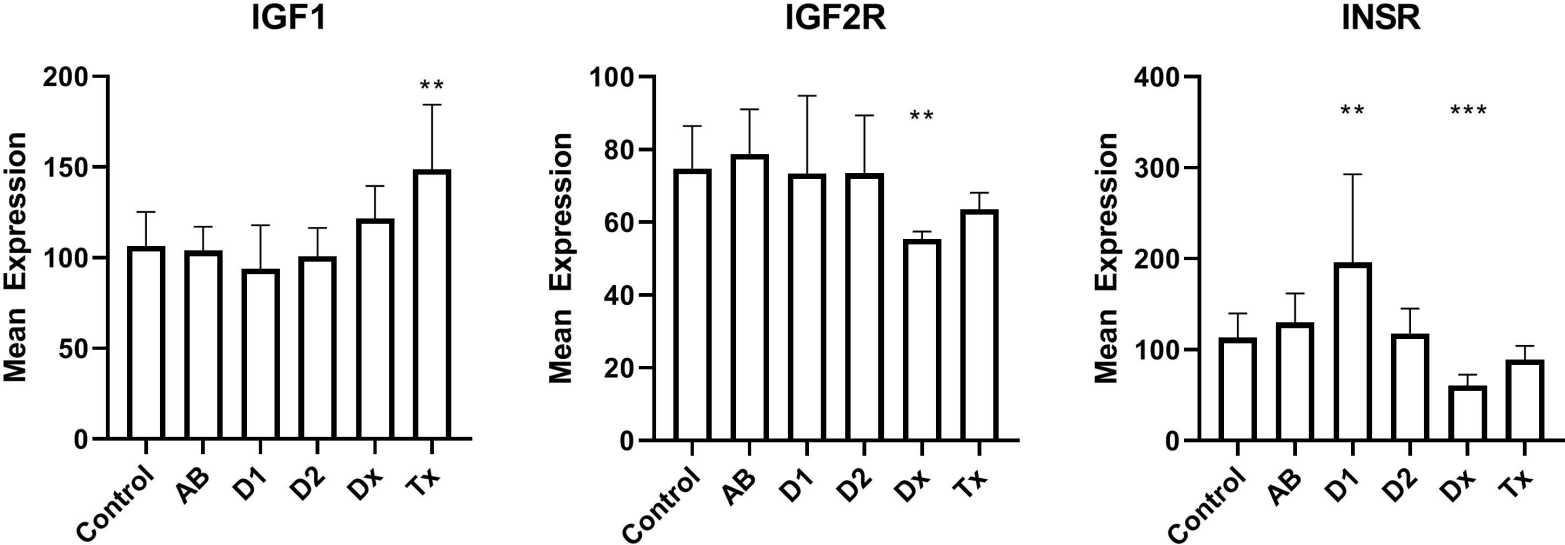
Islet mRNA expression of genes related to insulin action. Patient groups are identified in **Figure 1**, which also describes the statistical testing. *p<0.05, **p<0.01, ***p<0.001.

We finally examined the insulin receptors in the beta cells to exclude that changes in their expression could be a reason for dormant state. We found that mRNA for the insulin receptor gene was down-regulated by a factor of two in newly diagnosed T1D patients, but increased in tissue from long lasting T1D patients (**Figure 4**). Tissue from T2D patients did not show any change. Likewise, the IGF2 receptor gene expression is down-regulated to 66% of the normal value, but no changes are seen for IGF1 receptor.

We also looked for polymorphisms among all the investigated genes mentioned in **Table 2**, but found no significant polymorphisms.

## Discussion

In the present study we examined islets of Langerhans from live, newly diagnosed T1D humans and the results were compared to T1D patients with longer disease duration and other relevant controls. In the DiViD study enteroviruses were detected in all cases (22–24), and the inactive state of many beta cells could be a rational self-defense mechanism when infected with virus, as resting cells are less vulnerable than actively secreting cells; thus, enterovirus-infected mice with relaxed beta cells, induced by prophylactic insulin treatment, are less sensitive to diabetes development (25). The disadvantage of this inactivation state, however, is that the few remaining beta cells must be highly active and therefore are more vulnerable to immune attack. We therefore found it interesting to investigate the background for the dormant state.

We previously found that a number of genes related to the sphingomyelin synthesis were altered in T1D (21) and identified several additional genes in the present study. Based on expression levels of mRNA, it is not possible to predict changes in sphingomyelin levels, but a decrease in acid sphingomyelinase mRNA expression in tissue from all patient groups related to T1D (newly diagnosed, long standing, antibody-positive, transplantation tissue) compared to controls and T2D patients, is surprising, since decreased levels of acid sphingomyelinase is associated with higher levels of regulatory T cells (26), which is not expected in T1D-related conditions. For reference, increased sphingomyelinase has been found in serum from patients with rheumatoid arthritis (27).

We found decreased expression levels in five of six synaptotagmin mRNAs examined from T1D tissue and also in SYT16 expression from recently diagnosed T1D patients, still without detecting changes in tissue from T2D patients. While data are not available for all synaptotagmins in beta cells, SYT7 has previously been described as reduced in a beta cell model during cytokine stimulation (28) and further attributed to replenishing of insulin granules (29). Various data are available for SYT9, which we did not find altered, as it has both been described as a major Ca^++^ sensor (30), a regulator of early phase insulin secretion (31), and not involved in insulin secretion (32). SYT4 is decreased in tissue from T1D patients, which could be important, as an increase in SYT4 occurs during postnatal maturation of the beta cells, leading to binding of cellular Ca^++^ and resulting in a more specific glucose-stimulated insulin secretion. SYT11 and SYT13 were previously found reduced in human T2D islets (33), but not in the present study. In summary, the observed reductions in synaptotagmins could contribute to the diabetic phenotype. Although the SYT4 mRNA reduction is characteristic of undifferentiated, fetal beta cells, a fully, dedifferentiated state is unlikely, as it is not seen in adults (except in insulinomas), and since it is well known that fetal beta cells, exposed to glucose, as it takes place in pregnant T1D patients, develops into the adult phenotype.

As genes regulating the circadian rhythm are changed during hibernation (34), we used their expression to probe the hypothesis that the dormant state of the beta cells, found in human pancreatic T1D tissue, could be related to this condition. Beta cells display a distinct time-of-the-day circadian rhythm (35), so in principle the analysis is problematic, since the tissue is obtained from donors that deceased at different times of the day. If this was detrimental for the analysis, however, we would expect to see random expression levels of the circadian genes across the patient groups, and thus no significant differences. In contrast, we noticed a compelling number of circadian genes differentially regulated for T1D, either at diagnosis and/or in tissue from long-standing T1D patients, and significantly, only 2 genes that were differentially regulated in tissue from T2D patients. Regarding IL-6 we can not, of course, determine whether the signal arose from the beta cells or from infiltrating lymphocytes, but in any case, IL-6 was primarily included as a circadian gene, not as a marker of immunological activity. Together, we find that this suggests that the diabetic condition influences the expression of the circadian genes in the tissue collection. Their importance for insulin secretion is well established. Disruption of CLOCK and BMAL1 was previously shown cause to hypoinsulinaemia and diabetes in mice (36), and changes in the synchronization of the beta cells, resulting in loss of pulsatile or first phase insulin secretion, are predictive for both T1D and T2D (37, 38). Our data therefore support the hypothesis that desynchronized beta cells, also found during hibernation, are associated with the diabetes development.

We finally investigated the possibility that changes in beta cell insulin sensitivity could reduce the insulin secretion during diabetes development. We did see changes in insulin- and insulin-like growth factor 2-receptor expression in tissue from T1D patients (and not in tissue from T2D patients, as was also found by western blotting (39)), but it is not currently clear what the implication of the finding is, since it has been found that a lack of insulin stimulation on beta cells can both decrease (40) and increase (41) insulin stimulation. Interestingly, we and others have found that overweight adolescents, predisposed to T2D, also have a higher risk of developing T1D (42, 43). Furthermore, presence of insulin resistance can actually predict progression to T1D (44).

In summary, the human pancreatic beta cells that we investigated, show traits of embryonic dedifferentiation and changes in synchronization, similar to characteristics of T2D beta cells, and we previously found a relation between T1D development and predisposing factors to T2D disease. In this report, we primarily focused on cellular disease mechanisms, that could explain the inactivity of beta cells in T1D patients. If these mechanisms can be further established, it opens for prophylactic treatment of pre-T1D with GLP-1 analogues, metformin or potassium channel blockers to possibly activate the dormant beta cells. It could be done prophylactically in an early stage, following detection of T1D autoantibodies, and in combination with immunesuppressive medicine. Of note, GLP-1 improves glucose-stimulated insulin secretion and restores glucose competence in glucose-resistant beta-cells (45), and GLP-1 stimulation of SYT7 phosphorylation in pancreatic beta cells (46) might potentially overcome a decreased expression of the protein.

## Data availability statement

The raw data supporting the conclusions of this article will be made available by the authors, without undue reservation.

## Ethics statement

The studies involving human participants were reviewed and approved by The Norwegian government’s Regional Ethics Committee (reference 2009/1907). The patients/participants provided their written informed consent to participate in this study.

## Author contributions

KB conceived the idea behind the study of T2D mechanism and together with KJ arranged the various examinations and wrote the original manuscript draft. KD-J and LK performed the DiViD study. IG performed the RNA expression analysis. FP did the GWAS studies. All authors contributed to the article and approved the submitted version.
